# The “Hub and Spoke” (HandS) ECMO for “Resuscitating” Neonates with Respiratory Life-Threatening Conditions

**DOI:** 10.3390/children8010024

**Published:** 2021-01-05

**Authors:** Massimo A. Padalino, Nicoletta Doglioni, Daniel Nardo, Eugenio Baraldi, Vladimiro L. Vida, Daniele Trevisanuto

**Affiliations:** 1 Pediatric and Congenital Cardiac Surgery Unit, Department of Cardiac, Thoracic and Vascular Sciences, University of Padova, 35128 Padova, Italy; vladimiro.vida@unipd.it; 2 Neonatal Intensive Care Unit, Department of Woman’s and Child’s Health, University of Padova, 35122 Padova, Italy; nicoletta.doglioni@aopd.veneto.it (N.D.); daniel.nardo@aopd.veneto.it (D.N.); eugenio.baraldi@unipd.it (E.B.); daniele.trevisanuto@unipd.it (D.T.)

**Keywords:** extracorporeal membrane oxygenation, meconium aspiration syndrome, neonate, Hub and Spoke, outcome

## Abstract

Background: Extracorporeal membrane oxygenation (ECMO) implantation for neonates with severe cardiorespiratory life-threatening conditions is highly effective. However, since ECMO is a high-risk and complex therapy, this treatment is usually performed in centers with proven expertise. Methods: A retrospective review of neonates, from January 2014 to January 2020, presenting with life-threatening conditions and treated by means of Hub and Spoke (HandS) ECMO in peripheral (spoke) hospitals. Data were retrieved from our internal ECMO registry. Protocols and checklists were revised and shared with all spoke hospitals located in North-Eastern Italy. Results: Eleven neonates receiving maximal respiratory and cardiovascular support at a spoke hospital underwent HandS ECMO management. All but three patients were affected by life-threatening meconium aspiration syndrome (MAS). The median ECMO support duration and hospitalization were four (range 2–32) and 30 days (range 8–50), respectively. All but two patients (with congenital diaphragmatic hernia), were weaned off ECMO and discharged home. At a mean follow up of 33.7 ± 29.2 months, all survivors were alive and well, without medications, and normal somatic growth. All but one had normal neuropsychological development. Conclusion: HandS ECMO model for neonates with life-threatening conditions is effective and successful. A specialized multidisciplinary team and close cooperation between Hub and Spoke centers are essential for success.

## 1. Introduction

Extracorporeal membrane oxygenation (ECMO) can provide valuable life support for severe acute respiratory and circulatory failure. However, since ECMO is a high-risk and complex therapy, current literature suggests that this treatment should be performed in high-volume ECMO centers [1,2,3,4]. This is even more true in the pediatric field, where neonatal ECMO and appropriate surgical and technical expertise are available in very few tertiary hospitals [3].

As described elsewhere, conventional methods of transport of critically unstable patients may be unsafe or even dangerous [5,6,7]. However, a multidisciplinary team deployed from a high-volume ECMO center (hub) can effectively initiate ECMO at the referring (spoke) hospital and transport the patient on extracorporeal support back to the hub center [8].

Currently, neonates with acute respiratory distress (ARDS) who are unresponsive to conventional therapy can be treated successfully with ECMO [9,10]. Survival in meconium aspiration syndrome (MAS) after ECMO treatment is highly satisfactory [2,11]. However, about 10% of such neonates may need a prompt transfer to a highly specialized hospital for ECMO implantation [11,12,13].

On the basis of these facts, in 2014, we started a neonatal “Hub and Spoke” (HandS) ECMO program [14], intending to provide an ECMO service in multiple sites, where the “hub” was our ECMO center, and the “spokes” were the secondary connecting centers in North-Eastern Italy. Here, we report our seven years’ experience with neonatal HandS ECMO to evaluate results and outline safeguards and pitfalls.

## 2. Methods

This is a retrospective clinical analysis, including all neonates who required HandS ECMO support between January 2014 and January 2020. The study was approved by the Ethics Committee of the Azienda Ospedaliera-Università di Padova (ref. Prot n. 0021321/20.02.2020). Demographic and clinical data were collected from our institutional database. A follow-up assessment was performed by our neonatal intensive care physicians, including the Denver Developmental Screening Test revised (DDST-R), used for neurodevelopmental assessment at one year [15]. A descriptive analysis of data was performed, and continuous variables were expressed in mean ± SD, median, and range.

Of note, we evaluated our organization’s effectiveness and evolution by calculating our response times to calls from spokes hospital. There are multifactorial parameters, depending on the spoke center’s distance, climate/environmental issues, and spoke center competencies. Among these, the most relevant parameters were the following: team activation time (TAT), i.e., the time elapsed from the first alert call to leave from the hub) and ECMO initiation time (EIT), i.e., the time elapsed from the first alert call to the beginning of ECMO support).

### 2.1. Hub and Spoke (HandS) Extracorporeal Membrane Ooxygenation (ECMO) Organization

**Figure 1:**
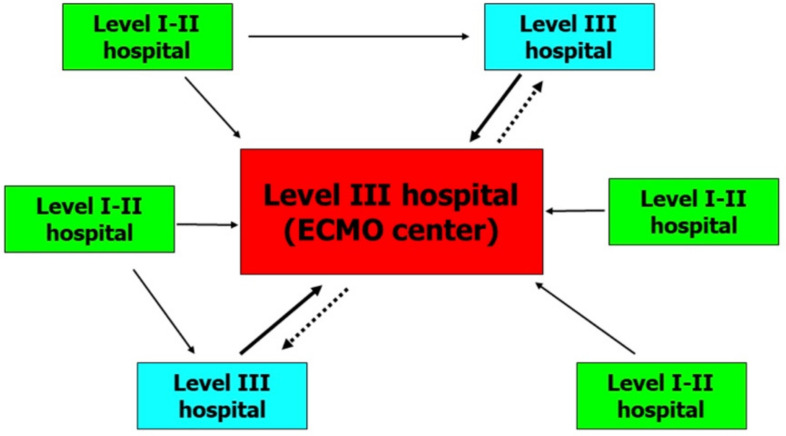
Organizational model for a regional extracorporeal membrane oxygenation (ECMO) network. Continuous lines indicate urgent transports and dashed lines indicate back transports.

Our neonatal health-care referral area is North-Eastern Italy (seven million people, with about 53,000 deliveries/year). The critical neonatal-and-pediatric transport program (CNaPTP) to spoke hospitals, created in 1999, has been utilized and easily adapted for HandS ECMO pediatric program. Our regional ECMO network includes patients admitted from level III spoke hospitals (Figure 1). Close cooperation between Hub and Spoke centers is encouraged (Supplementary Table S1) and spoke facilities usually repatriate their patients who have been weaned off ECMO and deliver the necessary long-term care to ensure a good turn-over at the hub center.

**Figure 2:**
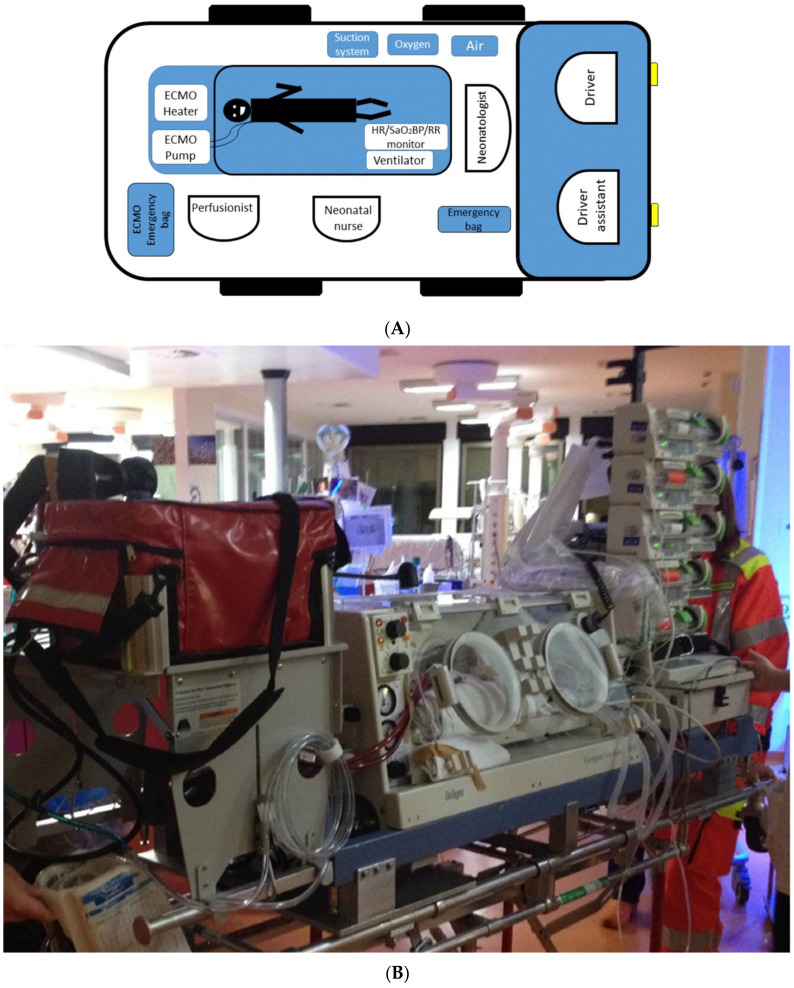
(**A**) Diagram showing allocation of the team members in relation to the patient and equipment positioning in the ambulance; (**B**) Picture showing our current setting.

An ECMO team is available 24/7 and includes seven members with ECMO expertise (two surgeons, a neonatologist, a perfusionist, a neonatal intensive care nurse, and two drivers, Table 1). An operating room scrub technician is asked to be provided by the spoke center. Last, a dedicated neonatal/pediatric ambulance and a second assisting vehicle are used for ECMO transports to allocate the patient, the team, and the equipment (Figure 2).

### 2.2. HandS ECMO by Steps

As we previously described [14], all HandS ECMO followed some precise steps, which were modified throughout the years, as we gained experience. These steps are as follows:

Step 1: Start at the hub

The HandS ECMO starts with the referral from the spoke center. A neonatal ECMO consulting hotline is active 24/7, and the patient is evaluated according to selection criteria for HandS ECMO candidates. These are derived from current international guidelines [7,16] and include severe cardiac and respiratory failure, refractory to maximal medical management, with a potentially reversible etiology, and oxygenation index (OI) > 40. Exclusion criteria from HandS ECMO were severe chromosomal or other lethal anomalies, irreversible pulmonary failure or brain damage, uncontrolled bleeding, grade III or greater intraventricular hemorrhage, low birth weight (<2.0 kg), and a distance from spoke center >200 km.

After the evaluation process, the decision to decline, accept a transfer, or activate our HandS ECMO team is made (Supplementary Table S2). If the patient is considered eligible for ECMO transport, the on-call HandS team and the ambulance drivers are alerted.

Step 2: Organization of transportation

After activation, ground transportation (2 vehicles for the patient, team, and ECMO supplies and materials) is arranged. Upon leaving the hub center, the patient’s conditions are monitored in real time with the requesting spoke center (Supplementary Table S3). At this point, the TAT is recorded.

Step 3: Spoke center cooperation

The Spoke Center is required to prepare the appropriate field (Supplementary Table S1) for a successful ECMO initiation, in the following 3 ways: (a) optimizing the patient clinical and hemodynamic conditions until the traveling team arrives; (b) providing an adequate surgical setting, such as clearing the right neck for cannulation, alerting the scrub technician, and procuring blood products for priming for the emergent ECMO implantation; (c) parental information and a complete description of risks and benefits of ECMO, to sort out all questions and doubts, and to obtain informed written consent.

Step 4: Arrival at the spoke center

Upon arrival, a final assessment of the patient’s clinical conditions is warranted to confirm ECMO indications’ persistence. Then, the patient is prepared on the bed in the upside-down position to have the neck well exposed and enough space to operate. After prepping the sterile field and surgically taking down of neck vessels (usually right carotid artery (RCA) and right jugular vein (RJV)), a veno-arterial ECMO is established in the usual fashion. At this point, the EIT is recorded (Supplementary Table S4).

Step 5: ECMO initiation and stabilization

ECMO support is started. Blood flow is gradually increased until the full flow is reached. A chest X-ray is performed to check the cannulas’ position. Inhaled nitric oxide and high-frequency oscillatory ventilation are weaned off and switched to conventional mechanical ventilation for transportation. Further therapeutic maneuvers include ventilator management during transfer (low ventilation settings to allow lung rest, PEEP 7–8 cmH2O, PIP 15–22), inotropes adjustment, and sedation with narcotic and benzodiazepine, while chemical paralysis is utilized only if the patient has a congenital diaphragmatic hernia (CDH).

Step 6: Transport and care at the hub

The patient is safely moved to the ground ambulance and transferred to the hub hospital for clinical management. In the ambulance, the positioning of the equipment and the team members is based on the specific roles and tasks (Figure 2).

When a successful transport to the hub center is achieved, and the patient is admitted into the ICU, a chest X-ray is usually repeated to check cannula position and rule out any cannulas’ dislocation during transportation. The following clinical care follows the treatment algorithms that we have previously described [17], developed by an internal multidisciplinary team, and modified according to technical improvement, recent guidelines, and all ECMO providers’ feedback. The weaning off ECMO is usually performed utilizing the so-called “bridge technique” [18], which, in our experience, has facilitated safe weaning despite the absence of a bedside specialist.

## 3. Results

From January 2014 to January 2020, 15 pediatric patients required HandS ECMO support in our center. Among them, 11 neonates with severe ARDS (MAS in eight neonates, CDH in two neonates, and salicylate poisoning in one neonate, Table 2) were referred from six different spoke centers in North-Eastern Italy. Of note, due to simultaneous requests, on one occasion, a second HandS ECMO team activation was required on the same day. Hub and Spoke distances ranged from 25 to 183 km (median 128), and we used ground transportation in all cases. The median TAT and EIT were 100 (30–150) and 105 (35–230) minutes, respectively. All patients underwent uneventful surgical neck vessel cannulation using pediatric 8 Fr arterial and 10 Fr venous cannula (Medtronic Inc, Minneapolis, MN, USA). After ECMO implantation and clinical stabilization, all patients were uneventfully transferred to our cardiac ICU, where nine patients were weaned off ECMO support without complications after a median time of four days (range 2–32). Soon after decannulation, patients were transferred to our neonatal intensive care unit for further care before repatriation or discharge.

One patient with ARDS caused by salicylate poisoning presented left hemisphere cerebral stroke two days after weaning off ECMO, due to occlusion of the left internal carotid artery, despite full anticoagulation during ECMO and after weaning. The precise cause was not identified. Full coagulation protein screening was within normal limits. The right carotid artery (where the arterial cannula was previously placed for ECMO cannulation) was partially patent.

Two patients with a severe degree of CDH (upward liver dislocation) did not survive. The first presented with severe pulmonary hypoplasia and was weaned off ECMO after 32 days and died immediately after compassionate care. After successful CDH repair and ECMO weaning, the other patient developed refractory pulmonary hypertension, was not responsive to pulmonary vasodilators, and required a redo ECMO, but died from retroperitoneal hemorrhage two days thereafter.

All remaining nine survivors were discharged from the hub after a median stay of 30 days (8–50); eight survivors were repatriated to the spoke center, while one was discharged home.

At a median follow-up of 14.4 months (range 1.3–74.8), all survivors were at home alive and well, with average growth and normal respiratory conditions. Neuropsychological development was normal in all but one patient, who had a stroke after ECMO, and is currently being treated with antiepileptic therapy. Postoperative data and Hands ECMO mission times are summarized in Table 3 and Table 4, respectively.

## 4. Discussion

Neonatal ARDS is a life-threatening condition that may require emergency respiratory ECMO support when conventional treatment options fail [9,10,11,12]. As these patients can fully recover if prompt treatment is established, it is essential to arrange a HandS ECMO service to provide assistance even in peripheral hospitals. Our experience has proven that this can be highly effective, with a 100% survival in neonates with severe MAS.

It is widely known that candidates of ECMO support have an estimated probability of death of 80–100%, despite maximal conventional therapies. According to the Extracorporeal Life Support Organization (ELSO) Registry [16], current ECMO survival rates are highly satisfactory, ranging from 41% in children with heart failure to 74% in newborns with any ARDS [16]. However, due to resource allocation and costs, specialized ECMO interventions are usually provided in tertiary, high-volume, dedicated centers with proven expertise that can provide the best care and optimize results [2,3,4,16,19,20].

The concept of a mobile ECMO team has been reported either for adults [1,5,7,8,11,19,20,21,22,23,24,25] and children [9,14,26], and highly successful transportation of patients on ECMO has been described for short and long distances by ambulance, helicopter, and airplane [25,27,28]. As stated by Coombs [1], “each ECMO network should ideally create mobile ECMO teams to retrieve patients and to deal with patients who have critical cardiopulmonary failure refractory to conventional therapy”. Hospital networks at the local, regional, or interregional level have been successfully created around tertiary referral hospital with ECMO expertise in the UK [22], Italy [26], and Australia [27] and have been associated with encouraging results for the treatment of the most severe forms of influenza A (H1N1)-associated ARDS [29].

Following this concept, in 2014, we started our HandS ECMO program, supported by the preexisting CNaPTP, which has been essential for success. The CNaPTP covers more than 20 hospitals in our institution and has the capacities and equipment (including HFOV, inhaled nitric oxide, and therapeutic hypothermia) to provide care to high-risk neonates during transportation.

The entire equipment, surgical technique, and management protocols during transportation and afterward do not differ from our standard. In our practice, we have been using our usual protocol modalities for managing ECMO, which are characterized by a limited number of human resources, as previously described [17]. Despite this “basic” arrangement, which has never reduced ECMO effectiveness [17], a successful organizational framework of the HandS ECMO model for neonates has been a viable and highly successful option for peripheral spoke hospitals, as demonstrated by the activation of two simultaneous teams.

Nonetheless, for this arrangement to succeed, it is essential that close cooperation between Hub and Spoke centers is established (Supplementary Table S1). In fact, in our experience, the spoke centers could achieve hemodynamic stabilization of these high-risk neonates and promptly recognized ECMO indication and timing. In order to do this, spoke hospitals in such a network should be trained and adhere to written standardized protocols that detail criteria for both the initiation of ECMO (indications and exclusions) [7,16], as well as optimization of conventional treatments to be undertaken before considering ECMO (such as low-volume, low-pressure, lung-protective ventilation or the use of prone positioning in patients with severe ARDS) [30]. Comprehensive plans regarding access to mobile ECMO should be created within networks. Referral centers and other network members should hold regular meetings to discuss network activity, including a review of ECMO cases. as well as those patients who were deemed inappropriate for ECMO. The preoperative stabilization and optimal timing, and multi-organ care of the patients are essential to minimize postoperative complications related to anticoagulation and ECMO circuit. One of our patients, who had been hemodynamically unstable and hypoxic for a more extended time than other patients, and experienced prolonged hypoxemia, had the only neurological complication we experienced in our series.

Furthermore, to ensure reasonable outcomes at the hub center, spoke facilities must repatriate patients and deliver the necessary long-term care. This kind of collaboration is vital and extremely rewarding because every medical center cannot offer the same degree of expertise in neonatal ECMO implants. However, the HandS model permits a tertiary institution to act as a hub center for peripheral hospitals without advanced cardiac or respiratory care, improving critically ill neonates’ survival.

When we deal with ECMO patients, timing is crucial; if too late, ECMO is useless. As practiced elsewhere [8], minimizing the time to reach and secure the patient is the main priority in planning the primary transportation. For this reason, we created some simple parameters (TAT and EIT) in order to evaluate our organization’s improvement and causes of failures, and that we expect to guide future decisions when a HandS ECMO program is growing. For this reason, the distance limit (<200 km) that we currently refer to may be modified in the future or even not applicable if using a different vehicle. In our experience, ground transportation was a more feasible and practical form of transport in our global organization. In addition, all ECMO transfers were uneventful, since we could benefit from the former CNaPTP experience. The potential risk of ECMO cannulas dislocation and the need to repositioning them during transportation remains possible but less probable when a highly specialized and expert team is provided.

Although the veno-venous (V-V) ECMO support is often indicated because of its simplicity and effectiveness in ARDS [31], in our experience, we have not used it, since ARDS can often significantly affect hemodynamic function in neonates. In addition, our emergency experience has been mostly with V-A ECMO rather than V-V ECMO, and we did not report increased complications as compared with the literature.

In Figure 1, we describe an organizational model for a regional ECMO network. All patients who received ECMO were admitted from level III hospitals where maximal cardiorespiratory support was already in place. Two out of 11 patients were born in a level I hospital and were transferred to level III hospitals unable to offer ECMO. About 12 h later, these critical patients met ECMO treatment criteria, and our HandS ECMO team was activated. This suggests that, although clinical conditions initially appear stable, it would be better for neonates with MAS to be quickly admitted to an ECMO center, because acute and rapid respiratory deterioration can occur during the first hours of life.

On the one hand, our experience has proven that HandS ECMO can be highly effective, with a 100% survival in neonates with severe MAS. The onset of post-ECMO complications in this series was not higher than that reported in the literature [16] and did not differ in quality from complications that could occur during standard ECMO management. On the other hand, in our series, HandS ECMO for CDH has been highly unsuccessful. Certainly, CDH is the first cause of neonatal ARDS requiring ECMO support [32] and causes pulmonary hypoplasia and hypertension, leading to cardiorespiratory failure with high mortality and long-term morbidity [33,34]. Survival, even with ECMO, is not more than 50% [35,36,37]. However, similar survival rates both with and without utilizing ECMO have been reported, and questions about the utility of ECMO support in the CDH population remain. Treatment of CDH is known to be difficult, and the analysis of repair outcomes on or off ECMO is prone to confounding factors, including variability in disease severity and overall management [36]. This supports our impression that there are no particular problems related to HandS ECMO support that affect the outcome. The severity of CDH and complicated management of postoperative pulmonary hypertension and prolonged ventilation-related complications may have played a significant role in the unsuccessful outcome. An anticipatory strategy should be considered in these cases. CDH may be considered to be an indication that the mother should be referred to a tertiary center with an ECMO facility to minimize additional perinatal lethal or invalidating complications for such a delicate neonate.

## 5. Conclusions

The HandS ECMO model for neonates with life-threatening conditions is effective and successful. Appropriate patient selection, proven ECMO and neonatal transportation expertise, and use of validated protocols and checklists, together with close cooperation among Hub and Spoke centers, are essential for success.

## Figures and Tables

**Table 1:** Personnel and roles of the ECMO team.

Personnel	Responsibilities
1 Neonatologist/pediatric intensivist	Patient and anesthesia management (i.e., ventilatory parameters, fluids, and inotropic infusion) before and after ECMO positioning, and during transfer
1 Pediatric cardiac surgeon	Patient management during surgical procedure, surgical positioning of the cannulae
1 Assistant surgeon	First surgical assistant
1 Perfusionist	Priming and management of the ECMO machine at implant and during transfer
1 Neonatal/pediatric nurse	Patient management during transfer, including monitoring, drug preparation
2 Drivers	Drive vehicles and arrange logistic aspects

**Table 2:** Patients characteristics at Hub and Spoke (HandS) ECMO implantation, and modes of ventilation before (at the spoke) and after (at the hub).

Patient	GA	BW	Diagnosis	OI	Surfactant	Spoke Care Level (1-2-3)	CS	Apgar Score 1′	Apgar Score 5′	Postnatal Age at ECMO (Hours)	Mode of Ventilation		FiO_2_		SatO_2_ (%)	
											At Spoke	At Hub	At Spoke	At Hub	At Spoke	At Hub
1	41	2786	MAS	30	YES	3	NO	4	6	10	HFOV	SIMV	1.00	0.30	60	100
2	40	2900	MAS	31	YES	3	YES	9	9	144	HFOV	SIMV	1.00	0.30	88	100
3	40	4280	MAS	90	YES	3	YES	3	5	48	HFOV	SIMV	1.00	0.30	84	99
4	41	3130	MAS	40	YES	3	NO	4	6	24	HFOV	SIMV	1.00	0.30	90	98
5	40	3100	MAS	40	YES	3	NO	1	2	6	HFOV	SIMV	1.00	0.80	100	100
6	40	3600	MAS	40	YES	3	YES	1	4	3	HFOV	SIMV	1.00	0.30	80	100
7	39	2900	Salicylate	40	YES	3	NO	8	9	240	HFOV	SIMV	1.00	0.40	55	100
8	40	3515	MAS	40	YES	3	YES	2	5	26	HFOV	SIMV	1.00	0.30	82	100
9	41	2760	CDH	30	YES	3	NO	7	7	12	HFOV	SIMV	1.00	0.21	95	100
10	40	3200	CDH	40	YES	3	NO	4	5	16	HFOV	SIMV	1.00	0.30	85	100
11	41	3100	MAS	42	YES	3	YES	4	5	25	HFOV	SIMV	1.00	0.21	60	98
*# Median (range)*	*40* *(39–41)*	*3100* *(2760–3600)*		*40* *(30–90)*		*3* *(3–3)*		*4* *(1–9)*	*5* *(2–9)*	*24* *(3–144)*			*1.00*	*0.30* *(0.21–0.80)*	*84* *(60–100)*	*100* *(98–100)*

Legend: BW, body weight; CS, cesarean section; CDH, congenital diaphragmatic hernia; GA, gestational age; MAS, meconium aspiration syndrome; OI, oxygenation index; SIMV, synchronized intermittent mandatory ventilation. # In the last row, in italics, we summarized the main data of this group of patients.

**Table 3:** Postoperative and follow-up data.

Postoperative	*n* (%)
Patients	11
ECMO V-A mode	11
ECMO support (d, median, range)	4 (2–32)
Hub ICU LOS (d, median, range)	6 (3–32)
Hub LOS (d, median, range)	30 (8–50)
Complications during HandS ECMO transfer	0 (0)
Weaning off	10 (90.9)
MAS (8)	8 (100
CDH (2)	1 (50) *
Salicilate intoxication (1)	1 (100)
Intensive care unit major complications	3 (27.3)
Stroke (salycilate intoxication)	1
Refractory pulmonary hypertension (CDH)	1
MOF (CDH)	1
Survival at discharge	9 (81.8)
MAS	8 (100)
CDH (2)	0 (0)
Salycilate intoxication (1)	1 (100)
Follow-up (months, median, range)	14.4 (1.3–74.8)
Survival at follow-up	9 (100)
Normal neurological status	8 (88.9)

Legend: CDH, congenital diaphragmatic hernia; ECMO, extracorporeal membrane oxygenation; ICU, intensive care unit; LOS, length of stay; MAS, meconium aspiration syndrome; VA, veno-arterial; VV, veno-venous. * One patient was weaned off for compassionate care.

**Table 4:** Times of HandS ECMO mission.

Patient	TAT (min)	EIT (min)	ECMO Cannulation Time (min)	Total ECMO Cannulation (min)	Time at Spoke (min)	Total Mission Time (min)	Distance (Km)
1	150	220	18	30	150	425	152
2	30	115	25	30	180	355	54
3	136	230	20	35	215	395	54
4	30	75	18	40	175	290	152
5	100	140	18	45	180	460	128
6	120	35	25	30	250	690	25
7	95	110	20	40	185	485	153
8	45	78	20	35	193	500	153
9	110	60	15	40	180	360	54
10	62	105	15	30	200	480	183
11	100	80	15	30	180	315	128
*median* *(range)*	*100* *(30–150)*	*105* *(35–230)*	*18* *(15–25)*	*35* *(30–40)*	*180* *(150–250)*	*425* *(315–690)*	*128* *(25–183)*

TAT, team activation time; EIT, ECMO initiation time; ECMO cannulation time, duration of surgical manouvre; total ECMO cannulation time, anesthesia induction + prepping of surgical field + ECMO surgical implantation; time at spoke, duration of stay at spoke center. In the last row, in italics, we summarized the main data of this group of patients.

## Data Availability

Data is contained within the article or Supplementary Material. The data presented in this study are available in Supplementary Material here.

## Supplementary Materials

The following are available online at https://www.mdpi.com/2227-9067/8/1/24/s1, Table S1: “SPOKE” CENTER ECMO CHECKLIST, Table S2: Pre-HandS demographic data form (for physisican), Table S3: Pediatric HandS ECMO transfer form (for Pediatric ICU nurse), Table S4: Pediatric HandS ECMO data form (for the pefusionist).

## Abbreviations

ECMO	Extracorporeal membrane oxygenation
HandS	Hub and Spoke
MAS	Meconium aspiration syndrome
ARDS	Acute respiratory distress
