# Supplementary material for: The “Hub and Spoke” (HandS) ECMO for “Resuscitating” Neonates with Respiratory Life-Threatening Conditions
Source: Children (Basel). 2021 Jan 5;8(1):24. doi: 10.3390/children8010024 (PMC7824856; doi:10.3390/children8010024)
Supplement: Supplementary file 1 [file children-08-00024-s001.pdf]

Supplementary Table S1. “SPOKE” CENTER ECMO CHECKLIST

- **<u>Order blood</u>** for ECMO circuit priming and for emergency (2 adults packed RBCs) O
- **Provide a <u>scrub nurse</u>** O
- **<u>Investigations</u> to be made**:

  Cranial ultrasound O

  Echocardiogram O

  Chest x-ray O

- **Recent <u>lab tests</u>**:

  Hb, WBC, Platelets, CRP, PCT, Alb, ionized Ca, Cr, Urea, Bilirubine O

  Lactate, APTT, prothrombin complex, Fibrinogen, D-dimer, Antithrombin O

  Arterial blood gas O

  Cardiac enzymes O

- **<u>Patient preparation</u>**:

  CVC (leave neck access free, available for ECMO implant) O

  Arterial -line O

  Securely fixed endotracheal tube O

  Indwelling catheter O

  Space to set up the ECMO system and sterile instrument back table O

  Height adjustable incubator with no matress O

Metal utility cart O

IV pole on casters O

External defibrillator (neonatal) O

- Alert in advance the **<u>radiology technician</u>** to be available for x-ray after implant O
- **<u>Relatives/parents</u>** should be available to obtain full informed consent O

Supplementary Table S2. Pre-HandS demographic data form (for physisican)

- Hub Center____________________ Spoke Center____________________
- Pt demographic
- Name_______________________ DOB_______________________
- Gender______________________Contact __________________
- Weight Gestational Age
- Reason for HandS Transfer
- Prematurity
- Respiratory failure
- RDS
- MAS
- PNX
- CDH
- Apnea
- Bronchioltis
- Other_________________________________
- Cardiac Failure
- Neurological disorder
- Surgical
- Other cause
- Level of severity of clinical conditions:
- Mild
- Moderate

Severe

Supplementary Table S3. Pediatric HandS ECMO transfer form (for Pediatric ICU nurse)

Parameter	At 1st call	At SPOKE	During transfer	At HUB
Cyanosis				
Icterus				
Paleness				
RDS				
Shock				
epilepsy				
Hypo/hyperactivity				
Urine output?				
Body Temperature (degrees C)				
HR				
RR				
Transcutaneous SatO2				
PA				
Htc %				
pH				
pO2				
pCO2				
HCO3				
ABE				
FiO2				
Mechanical ventilation				
CPAP				
Intubation AMBU vent				
Intubation mech Vent.				
PI/PEEP				
RR (if mechanical vent)				
Tidal				
CXR				

Echocardiography				
Cerebral US				
Lines<br>Vein<br>Umbelical artery<br>NG tube				
Chest tube				
Medication list				
PGE1				
Surfactant done?				
CPR ?				

Supplementary Table S4. Pediatric HandS ECMO data form (for the pefusionist)

- Hub Center____________________ Spoke Center____________________

- Pt demographic
    - First and Last Name____
    - DOB_______________________ Gestational Age_________________
    - Gender______________________ BSA ___________________________
    - Weight ________________Height _______________

- Reason for HandS Transfer: Diagnosis________________________________
    - Prematurity
    - Respiratory failure
        - RDS
        - MAS
        - PNX
        - CDH
        - Apnea
        - Bronchioltis
        - Other_________________________________
    - Cardiac Failure
    - Neurological disorder
    - Surgical
    - Other cause

- ECMO details
    - Pump type____
    - ECMO circuit type ____
    - Oxygenator type ____

- Site of arterial cannulation ____________________________________
- Type or arterial cannula (size)_________________________________
- Site of venous cannulation____________________________________
- Type of venous cannula (size)_________________________________
- Type of Priming____
- Flow (l/min)_______________
- Cannulation time(min)_______
- TAT (min)___________________EIT (min)______________________

- Wean off? y/n __________________________ECMO duration (days)__________
- Date of end ECMO______________________________________
- Outcome: alive/dead
- Complications:
- None
- Hemorrhage/thrombosis
- Cannula dislocation
- Stroke
- Infection
- Change of ECMO: y/n
- Other
